# Diagnostic Dilemma of Cardiac Syncope in Pediatric Patients

**Published:** 2008-02-01

**Authors:** Ranya A Hegazy, Wael N Lofty, Rasha I Ammar, Aya M Fattouh

**Affiliations:** Department of Pediatrics, Faculty of Medicine, Cairo University

**Keywords:** Pediatric, cardiac, syncope

## Abstract

**Aims:**

Syncope is defined as temporary loss of consciousness and postural tone resulting from an abrupt transient decrease in cerebral blood flow. The present work aimed at determining how diagnostic tests are used in the evaluation of pediatric syncope at a tertiary pediatric referral center and to report on the utility and the yield of these tests.

**Settings and Design:**

Retrospective study conducted at a tertiary referral arrhythmolology service

**Methods and Material:**

The clinical charts of 234 pediatric patients presenting with a primary complaint of syncope with an average age of 7.48 ± 3.82(3.5-16) years were reviewed by the investigators.

**Statistical analysis used:**

Statistical Package of social science (SPSS) version 9,0 was used for analysis of data.

**Results:**

The commonest trigger for syncope in the study population was early following exercise (n=65) and the commonest prodrome was palpitation, noted in 25 patients. A murmur was present in 19 of our patients (8.3%) while  10.7% (n=25) had abnormal ECGs. Of the 106 echocardiograms done, 14 (13.2%) were  abnormal. Only two of them were missed by ECG. All patients were offered ambulatory 24 hour ECG. One patient with sick sinus syndrome was diagnosed only with Holter.

**Conclusions:**

Clues to the presence of cardiac syncope may include acute onset of syncope, frequent episodes, low difference between blood pressure readings in supine and erect positions (after standing for 2 minutes) and most importantly an abnormal 12 lead ECG. Transthoracic echo and Holter monitoring have low yield in pediatric syncope.

## Introduction

Syncope is defined as temporary loss of consciousness and postural tone resulting from an abrupt transient decrease in cerebral blood flow [1]. It's a relatively frequent symptom in children and its evaluation is an important aspect in pediatric medical practice [2]. Although the incidence of pediatric syncope is difficult to assess, it appears to peak around the age of 15 years, with 20% to 50% of females reporting to have experienced at least one syncopal episode by the age of 20 years [3].

Whereas the vast majority of episodes of syncope are benign, a minority are caused by something potentially more serious or even life threatening [4]. Even benign syncopal episodes may have dramatic presentations [5]. Family members and primary care providers may suspect a malignant cardiac condition prompting referral to pediatric cardiologists [6]. Medical evaluation of pediatric syncope may be expensive, frustrating and often with low yield [7]. The present work aims at determining how diagnostic tests are used in the evaluation of pediatric syncope at a tertiary pediatric referral center and to report on the utility and the yield of these tests.

## Patients and methods

The clinical charts of all pediatric patients presenting to the arrhythmology service, department of Pediatrics, Faculty of Medicine, Cairo University in the time period between June 2001 and January 2006, with a primary complaint of syncope were reviewed by the investigators. Syncope was defined as sudden, brief and complete loss of consciousness.

Witnessed as well as unwitnessed events were included. Patients with even a single episode of syncope were eligible for the study.

### Data collected from the medical charts included

#### History of syncopal episode:

- Frequency of episodes, triggers of syncope (prolonged standing, hyperventilation, emotions, fasting, exercise and others) and any aura prior to the event (dizziness, palpitation, decreased vision, nausea, sweating and pallor).
- Medications used by the patient.
- Family history of syncope, sudden death and  cardiovascular disease.

#### Examination of the patient:

Heart rate, diastolic and systolic blood pressure in supine and erect positions (after standing for 2 minutes). The presence of any murmurs or cardiovascular abnormalities was noted.

#### Routine 12 lead ECG was performed in all patients:

All routine measurements were taken during sinus rhythm. Data collected from ECGs included rhythm, heart rate, P wave amplitude, duration, QRS duration and axis, RV1, RV6,SV1, SV6 and QTc.

#### Twenty-four hour ambulatory ECG (Holter) recording:

Digital recording with ≥ 20 hours of artifact free data were included for all subjects. Data was analysed using the VisionTM  Holter analysis software system with manual editing and reviewing of all data. The mean, maximum and minimum heart rates, pauses (defined longer than 2 seconds), supraventricular and ventricular ectopics were recorded.

#### Transthoracic Doppler echocardiographic examinations:

These were carried out in patients where potential cardiac disease was suspected by history, examination or 12 lead ECG. The equipment mostly  used for all Doppler echocardiographic examinations was Hewelett Packard (Sonos- 4500), using an 8 and 4 MHz transducer, capable of performing 2-D studies, continuous and pulsed Doppler and color flow Doppler.

The echocardiographic measurements were made in accordance with the norms suggested by the American Society of Echocardiography [8]. The images for 2-D studies were also obtained according to the usual standardization [9].

#### Transthoracic Doppler echocardiographic examinations:

Some elements suggestive of potential cardiac disease included [10]:

- Prior personal history of fatigue, exercise intolerance, known arrhythmia or heart disease.
- Syncope preceded by palpitations / chest pain.
- Syncope during exercise
- Syncope without prodrome

Head upright tit table (HUTT) test is not available at our center and hence was not included as a routine work up for our study population.

## Statistical Methods

Statistical Package of social science (SPSS) version 9,0 was used for analysis of data. Data was summarized as mean, SD and frequency. T- test was used for analysis of two independent quantitative variables. While Chi - square test was used for analysis of qualitative data. P-value was considered significant if < 0.05.

## Results

234 patients were enrolled in the present work with an average age of 7.48 ± 3.82 years with a range of 3.5-16 years. Patients had an average number of 4 syncopal episodes prior to presentation with a range of 1 - 13 episodes. The study included 120 females (51.2%) and 114 males (48.8%).

The history of the study group showed the following: 170 patients (72.5%) described their syncope as being of acute onset while the remaining patients described a more gradual onset. 152 patients (65.2%) had recurrent episodes of syncope.

Triggers of syncope among our study population are shown in Table 1, the commonest being early following exercise noted in 65 (27.7%) patients.

Palpitation was the commonest prodrome among our study population (10.6%), but patients also complained of nausea (9%), excessive sweating (4.2%), dizziness (3.8%) and spots before eyes in 2%. 75 (32%) had no prodrome prior to their syncope. Family history for syncope was positive in 7% of patients. None had family history of cardiac disease or sudden death.

Examination of our patients showed that they had mean heart rates of 102 BPM ± 16.79 BPM with a range of 65- 160 BPM. They had a mean systolic BP during standing of 102 mmHg ± 15.8 with a range of 72-145 mmHg, while during sitting was 104.8 mmHg ± 13.8 mmHg with a range of 74 - 145 mmHg. Diastolic blood pressure on standing was 64.7  ± 10.6 mmHg with a range of 41 ± 99 mmHg while on sitting was 61.5  ± 9.5 mmHg with a range of 44- 90 mmHg. The differences between readings in supine and erect (after standing for 2 minutes) positions of both systolic and diastolic blood pressures are shown in Table 2. 19 of our patients (8.3%) had murmurs on examination. All patients underwent routine 12 lead ECG. Their measurements are shown in Table 3.

Abnormal ECG's were present in 10.7% (n=25). Abnormalities were in the form of preexcitation in 4, LQTc in 3, voltage criteria of ventricular hypertrophy in 8, atrial enlargement in 2, abnormal axis in 3, complete heart block in 2, supraventricular tachycardia in 2 and one patient with frequent ventricular couplets and a single transient run. Criteria of syncope were compared among patients with normal and abnormal ECG (Table 4).

None of them was found to be statistically significantly different except the onset of syncope and frequency of syncopal episodes. This was evident by the fact that all patients with abnormal ECGs described their syncope as being acute, in contrast to an acute onset being present in only 69.2% of those with a normal ECG (p=0.01). Frequent episodes of syncope were seen in  92% of those with abnormal ECGs as compared to only 61.9% of those with normal ECGs (p=0.04).

Comparison of the heart rate and blood pressure in patients with normal and abnormal ECGs is shown in Table 5.

Patients with abnormal ECGs had significantly higher diastolic blood pressure in erect position and lower differences in erect and supine positions of both systolic and diastolic blood pressures.

Patients with abnormal ECGs and those with history or examination suggestive of a cardiac problem were offered transthoracic echocardiography.

A total of 106 patients had echocardiographies done. 14 of those (13.2%) were found abnormal. These abnormalities were as follows: 4 dilated cardiomyopathy, 2 HOCM, 2 mitral valve prolapse, 2 ventricular septal defects, 2 rheumatic mitral valve regurge, 1 aortic stenosis and 1 atrial septal defect.

Two of these patients had normal 12 lead ECGs, one with mitral valve prolapse and one with rheumatic mitral valve regurge. Criteria of syncope as regards triggers and premonitory symptoms were compared among patients with normal and abnormal echocardiographies, however none was found different.

Importantly, those with abnormal echocardiographies had a significantly high correlation to abnormal ECGs (p= 0.0001) (Table 6)

All patients had a 3 channel 24 hour ambulatory ECG done. The results of their Holter recordings are shown in the following table (Table 7).

Patients who were diagnosed with cardiac abnormalities based on their 12 lead ECG or echocardiography summed up to 27 patients. Our study group was hence classified into cardiac and non cardiac and the Holter recordings of both groups were compared. (Table 8)

Holter recordings were not significantly different among those with and those without cardiac abnormality. One patient who had a normal ECG and echo was diagnosed as sick sinus syndrome from his ambulatory ECG recording. He was eight years old complaining of recurrent episodes of exercise induced syncope.

## Discussion

Syncope is a common event in the children that may cause great anxiety in parents, teachers and peers. It usually leads to frequent visits to pediatricians and emergency departments. It is caused by a wide array of etiologies that include abnormalities of the autonomic nervous system, as well as cardiac, neurologic, psychogenic and metabolic problems [11]. Consequently, an evaluation that fails to approach this medical problem in a goal oriented fashion may be expensive, time consuming and frustrating to all concerned. For every patient, an initial goal is to determine if an uncommon but serious form of cardiac disease is present.

The present work diagnosed a cardiac cause for syncope in 11.5% of children who presented to our tertiary referral center. This is somewhat similar to that reported by Driscoll et al [12](10%), yet discrepancies with other studies is also notable. Lower contribution of cardiac causes to pediatric syncope were reported by others (4.5%) [13], (3.9 %) [7] and (2 %) [11] and much higher percentages were reported by Kilic et al, [2](30.5%) and Wolff et al, [14] (28%).

Upon reviewing the attribution of cardiac causes to syncope in children, one must put in mind the sample of population used in that study. Ours is recruited from a tertiary referral center which might explain the higher percentage of diagnosed cardiac problems than those where samples were recruited from emergency departments [11] or primary care centers [7]. Another point to be considered is the definition of cardiac syncope. In a study by Ritter et al, [13] 35 patients diagnosed with minor valve lesions, PDA, septal defects and decreased left ventricular shortening fractions were not included among those diagnosed with cardiac syncope. They included patients with cardiomyopathies and cardiac electrical disorders only. However in our work all patients with abnormal echocardiograms were considered suffering cardiac syncope which may have raised their contributing percentage to syncope etiology. We believe that patients with abnormal cardiac lesions suffering syncope warrant a closer follow up and management, than those with normal hearts.

The importance of thorough history taking and physical examination in the evaluation of pediatric syncope has been stressed by many authors [11,15,16]. However the present work found that history and examination alone were not sensitive enough to differentiate cardiac from non cardiac causes which is in agreement with Ritter et al, [13]. Children are usually not accurate in recounting  their syncopal episode and prodrome; moreover many of these occurred at school and thus were not witnessed by an available source. In contrast to a previous study, [11] we did not find the presence of cardiac murmurs to correlate significantly with the presence of cardiac cause for syncope.

Interestingly, we observed that patients who were diagnosed with noncardiac syncope had significantly higher diastolic blood pressure on standing and lower differences between erect and supine (after ten minutes) readings of both diastolic and systolic blood pressure. This is explained by the fact that patients with neurocardiogenic syncope, the most common cause of pediatric syncope [6,10,11] have abnormal response to orthostatic stress.

Doing a 12 lead ECG as an initial step in the evaluation of syncope is extremely valuable in the diagnosis of a cardiac aetiology [1,10,12,17]. It has diagnostic and prognostic value in the evaluation of syncope, and consensus guidelines suggest routine electrocardiographic testing [18] . In the present study, an abnormal ECG was found in 25 (10.7%) of patients and was a primary clue of cardiac abnormality in these patients.  A 12 lead ECG didn't miss any of the major cardiac abnormalities in our patient group and therefore we strongly recommend that an ECG be used in evaluation of syncopal episode in children.  In our study, the yield of ECG was higher than that reported by others [7,19,20].

Cardiac arrhythmias should be considered among the malignant causes of syncope [2,21]. In the present study, arrhythmias were diagnosed in 13 patients (5.5%). Among those, 12 lead ECG was diagnostic in 12 patients and 24 hour ambulatory ECG diagnosed one patient with sick sinus syndrome. Kilic et al, [2] reported arrhythmias as a cause of 30.5% of cases of pediatric syncope. This may be explained by the much higher selectivity of patients in this study that can't be taken as a representative of the pediatric population. Ambulatory ECG showed no significant difference in findings (ventricular ectopics, supraventricular ectopics, respiratory sinus arrhythmia, first degree heart block, pauses) among both cardiac and non cardiac patients. Despite the low yield of ambulatory 24 hour ECG, 28 patients reported the occurrence of syncope on the day of the recording. Detecting a sinus rhythm during the episode effectively excluded arrhythmias as a cause of syncope. However, the attacks have to occur with a frequency that allows capture during the 24 hour recording. Advising patients and parents to stimulate triggering events and keeping a diary may increase yield of Holter ECG. In the present work, Holter diagnosed only one patient missed by 12 lead ECG, however his symptoms were suggestive of arrhythmias as being frequent and exercise induced. Although we performed Holter recording in all patients presenting with syncope, this may be considered a waste of time and effort. Limiting ambulatory ECG to patients with abnormal 12 lead ECG, frequent symptoms [1], positive family history and exercise induced syncope6 may lead to higher yield of ambulatory ECG and make the effort expended worth while. Palpitations and chest pain associated with syncope have been proposed as clues for arrhythmia induced syncope [6,10], however no such correlation as found in the present work.

Echocardiography was performed in 106 patients and only 14 (13.2%) patients had a cardiac anomaly. Of these, only two patients (one with mitral valve prolapse and the other with rheumatic mitral regurge) were missed by 12 lead ECG. This yield is higher than that reported by others [7,13]. In the study by Kilic et al, [2] it was recommended that echocardiography should be a part of the initial work up of pediatric syncope. We however don't support this notion. We like others [7,13] recommend that echocardiography should only be done in patients with abnormal 12 lead ECG. It may also be recommended in patients with normal ECG but positive family history, exercise induced syncope or in association with fatigue and exercise intolerance.

## Conclusion

Syncope is a common problem in pediatric medical practice. A consensus for diagnosis in a goal oriented approach is essential. A thorough history, physical examination and 12 lead ECG are highly sensitive primary lines of investigation to exclude cardiac causes of syncope in children. If an abnormality is diagnosed or suspected, then more sophisticated and time consuming investigations as transthoracic echocardiography and 24 hour ambulatory ECG monitoring are warranted. Clues to the presence of cardiac syncope may include acute onset of syncope, frequent episodes, low difference between blood pressure readings in supine and erect positions (after standing for 2 minutes) and abnormal 12 lead ECG.

## Figures and Tables

**Table 1:**
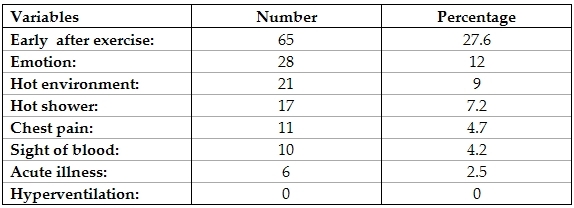
Frequency of triggers  of syncope of patients included in the study

**Table 2:**
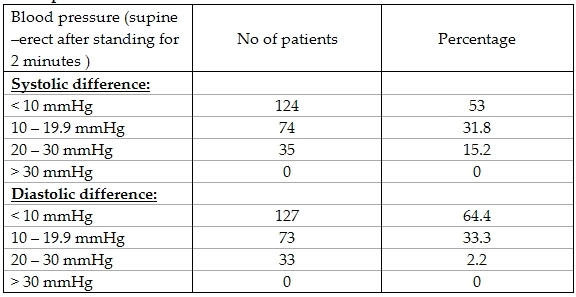
The differences between readings in supine and erect (after standing for 10 minutes) positions of both systolic and diastolic blood pressures

**Table 3:**
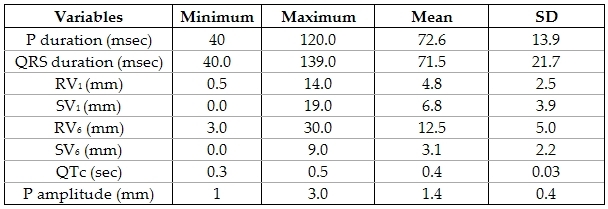
Descriptive statistics of ECG measurements of the study group

**Table 4:**
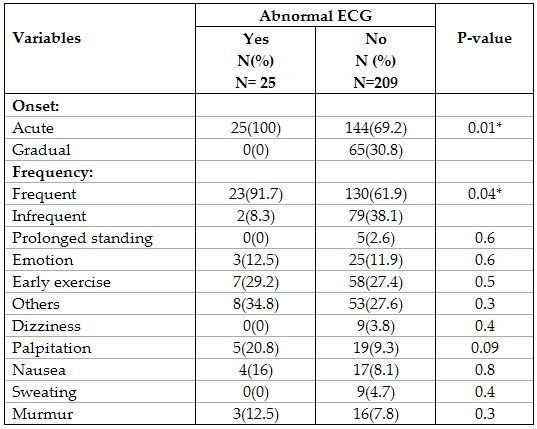
Comparing criteria of syncope in those with and without abnormal ECG

**Table 5:**
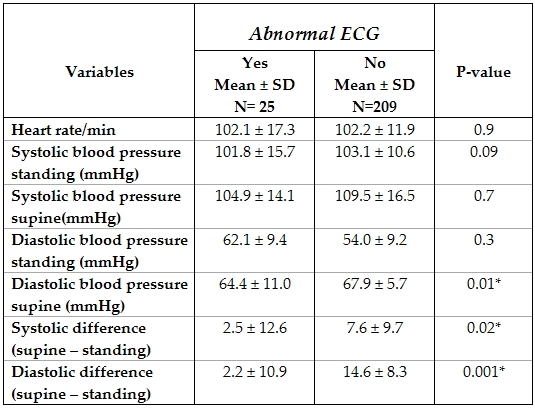
Comparing criteria of heart rate and blood pressure among  those with  and without abnormal ECG

**Table 6:**
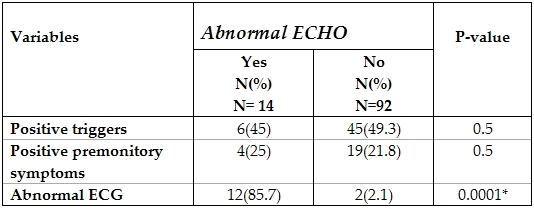
Comparing criteria of syncope in patients with and without abnormal ECHO

**Table 7:**
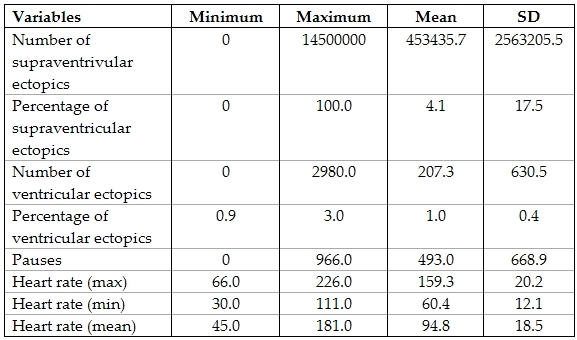
Descriptive statistics of results of 24 hours ECG in the study group

**Table 8:**
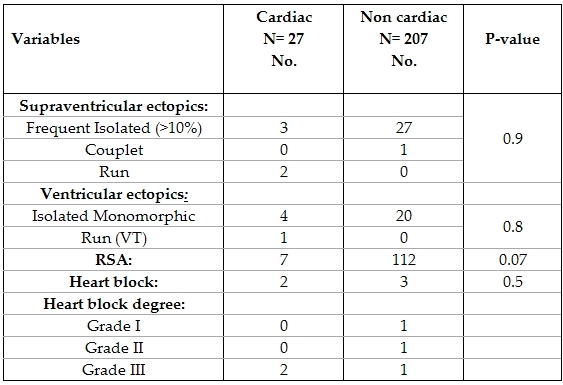
Holter ECG among cardiac and non cardiac  patients
